# Long-term variations in solar radiation, diffuse radiation, and diffuse radiation fraction caused by aerosols in China during 1961–2016

**DOI:** 10.1371/journal.pone.0250376

**Published:** 2021-05-03

**Authors:** Hongfei Xie, Junfang Zhao, Kaili Wang, Huiwen Peng

**Affiliations:** 1 State Key Laboratory of Severe Weather, Chinese Academy of Meteorological Sciences, Beijing, China; 2 Resources College, Sichuan Agricultural University, Chengdu, China; Institute for Advanced Sustainability Studies, GERMANY

## Abstract

The effects of atmospheric aerosols on the terrestrial climate system are more regional than those of greenhouse gases, which are more global. Thus, it is necessary to examine the typical regional effects of how aerosols affect solar radiation in order to develop a more comprehensive understanding. In this study, we used global AErosol RObotic NETwork (AERONET) data and robust radiation observational evidence to investigate the impact of aerosols on total radiation, diffuse radiation, and the diffuse radiation fraction in China from 1961 to 2016. Our results showed that there were different temporal changes in the aerosol optical depth (AOD), total solar radiation, diffuse radiation and diffuse radiation fraction over the past 56 years. Specifically, the 550 nm AOD from 2005 to 2016 decreased significantly, with annual average AOD of 0.51. Meanwhile, the average total solar radiation reduced by 2.48%, while there was a slight increase in average diffuse radiation at a rate of 3.10 MJ·m^-2^·yr^-1^. Moreover, the spatial heterogeneities of AOD, total radiation, diffuse radiation, and the diffuse radiation fraction in China were significant. Aerosol particle emissions in the developed eastern and southern regions of China were more severe than those in the western regions, resulting in higher total radiation and diffuse radiation in the western plateau than in the eastern plain. In addition, aerosols were found to have negative effects on total radiation and sunshine hours, and positive effects on diffuse radiation and diffuse radiation fraction. Further, the diffuse radiation fraction was negatively correlated with sunshine hours. However, there was a positive correlation between AOD and sunshine hours. These results could be used to assess the impacts of climate change on terrestrial ecosystem productivity and carbon budgets.

## Introduction

The increasing burden of aerosol particles in the atmosphere poses a serious threat to both human health and the climate [1]. Aerosols are gaseous dispersion systems composed of solid or liquid particles suspended in the gas medium, and can indirectly and directly alter the Earth’s radiation budget and global or regional climates, and thus play an important role in the Earth’s climate system and hydrological cycle [2, 3].

The most important impact of aerosols on climate is radiation. An increase in aerosol concentration reduces the total radiation received by the Earth’s surface. The fifth IPCC assessment report stated that the global radiative forcing caused by aerosols, and the clouds and black carbon since the industrial revolution reached -0.9 W·m^-2^ from 1750 to 2011 [2]. Therefore, with increasing aerosol concentrations in the atmosphere, the solar radiation reaching the Earth’s surface continues to decrease. Similar conclusions have been obtained in both observational experiments and model simulations. Regarding observations, the Indian ocean aerosol observation experiment found that the amount of solar radiation reaching the Earth’s surface decreased significantly with increasing absorbing aerosol concentrations [4]. Model simulations had also indicated that aerosol radiative forcing in China was between -13 W·m^-2^ and -5.3 W·m^-2^, in which the radiative forcing in autumn and winter was significantly less than that in spring and summer [5]. With increasing aerosol concentration, the total radiation during the maize growing season in China reduced by 13%, and the total radiation in the Northeast China, northern China, and eastern China decreased by 11.4%, 17.1%, and 15.6%, respectively [6]. In addition, solar radiation caused by aerosols in highly polluted areas, such as the North China Plain, the middle and lower reaches of the Yangtze River, east China, and the Sichuan Basin, decreased by 28%-49% [7].

Increases in aerosol concentration also increase the proportion of diffuse radiation. Atmospheric aerosols, especially the 0.01–10 μm aerodynamic diameters aerosols particles, can seriously affect cloud formations and increase the ratio between radiation and total photosynthetically active radiation (f_diff_) in the atmosphere [8]. This is especially relevant for high-latitude ecosystems, which are already exposed to a higher f_diff_ because of their solar height and high frequencies of overcast and cloudy conditions [9]. In addition, aerosols simultaneously weaken direct radiation and enhance diffuse radiation by absorbing, reflecting, and scattering solar light, thus altering the partitioning of direct and diffuse radiation by increasing diffuse fraction [4, 10]. These behaviours have been confirmed by observation and model simulation results [2, 4, 11–14]. For instance, Xue et al. [14] pointed out that seven regions in China from 2001 to 2014 had increases in diffuse radiation, with the fastest rate of 0.34 W·m^-2^·yr^-1^ occurring in central China. Meanwhile, the slowest rate of 0.04 W·m^-2^·yr^-1^ occurred in southern China.

Globally, China is one of the major regions suffering from substantial aerosol haze pollution [15]. In recent decades, with the China’s rapid development of urbanisation and industrialisation, a lot of aerosols have been released into the atmosphere, causing serious air pollution and radiation changes in China [16]. In the twenty-first century, aerosol pollution became an urgent national crisis in China [14]. Many studies have focused on long-term spatiotemporal radiation variations in China in order to evaluate the trends and influencing mechanisms of radiation [17, 18]. Some studies have argued that atmospheric aerosol variation is mainly responsible for variations in solar radiation, and a process of dimming (1961–1989) to brightening (1989–2013) in China as a result of aerosol changes was discovered [19, 20]. However, some researchers have drawn discordant conclusions. Zhou et al. [21] suggested a downward trend of surface radiation greater than 20 MJ·m^-2^ decade among five different climatic zones in China from 1962 to 2015. Ren et al. [22] observed a higher diffuse radiation fraction caused by aerosol pollution in China. Nevertheless, most studies were based on site-measured radiation datasets, and serious spatial discontinuities and low spatial coverage limited the adequate understanding of radiation in China. In addition, the effect of atmospheric aerosols on the terrestrial climate system is more regional than that of greenhouse gases, which are more global [23]. Thus, in order to develop a more comprehensive understanding, studying the typical regional effects of aerosols on solar radiation, diffuse radiation, and diffuse radiation fraction in China are very necessary.

The objectives of this study were to: (1) exploring the spatiotemporal variations of AOD, total radiation, diffuse radiation, and diffuse radiation fraction in China using global AERONET data and robust radiation observational evidence; (2) investigate the impacts of aerosols on total radiation, diffuse radiation, and diffuse radiation fraction in China from 1961 to 2016 using long-term datasets; and (3) provide scientific support for researching the impacts of climate change on ecological environment in China. These findings will be of use to regional policymakers.

## Data and methods

### AErosol RObotic NETwork (AERONET)

**Fig 1:**
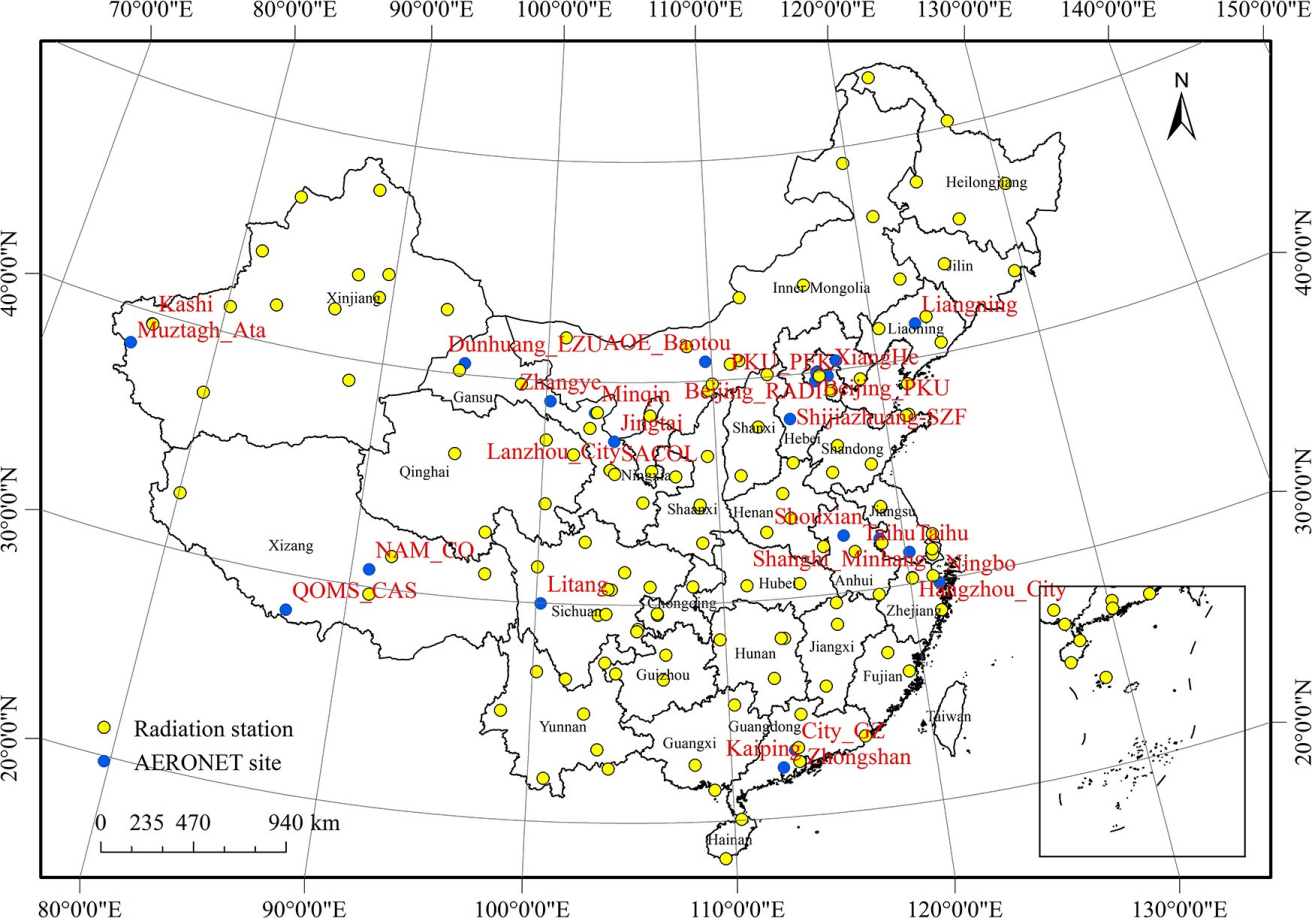
Distribution of the AERONET site and radiation station in China selected in this study.

The AERONET collaboration with high temporal resolution uses a network of ground-based sun photometers that provide global long-term and continuous measurements of aerosol optical, microphysical, and radiative properties, including parameters of AOD, Angstrom exponent, and aerosol inversion products [24]. These data provide level 1.0, level 1.5, and level 2.0 three accuracy levels. Level 1.0 and level 1.5 indicate not screened and screened for clouds. Level 2.0 represents screened for clouds and quality guaranteed, which contain AOD values in the channel. The processing algorithms of AERONET have evolved from Version 1.0 to Version 2.0 and are now on Version 3.0 [25]. Level 2.0 AOD monthly data from AERONET (version 3.0) in the 440 nm channel was used for this study. The AOD at 550 nm was calculated based on the AOD at 440 nm. In this study, 34 typical AERONET stations in China were selected for comparison (Fig 1). As a result, we found that the AOD data before 2004 were not complete for each state. Thus, we used the 550nm AOD data from 2005 to 2016 for each AERONET station as they had more comprehensive data to analyse the spatiotemporal evolution trend of the AOD.

### Observation data

Daily meteorological observations between 1961 and 2016 for 127 stations were provided by the National Meteorological Information Centre, China and were entered into a database containing the following variables: daily solar total radiation (MJ·m^-2^·d^-1^) and daily diffuse radiation (MJ·m^-2^·d^-1^) (Fig 1). We calculated the trends and variations in total solar radiation, diffuse radiation, and diffuse radiation fraction from 1961 to 2016.

### AOD

AOD is one of the most important parameters to determine the atmospheric turbidity. Generally, high AOD values indicate the increase of aerosol vertical accumulation, which lead to the decrease of atmospheric visibility. The AOD derived from AERONET in the 550 nm channel was calculated using the following [24]:

$$\frac{\tau_{\lambda_{1}}}{\tau_{\lambda_{2}}}={\left(\frac{\lambda_{1}}{\lambda_{2}}\right)}^{{-\alpha }_{\lambda_{1}-\lambda_{2}}}$$ (1)

where $\tau_{\lambda_{1}}$ and $\tau_{\lambda_{2}}$ represent the aerosol optical depth at *λ*_1_ and *λ*_2_ bands, respectively; *λ*_1_ and *λ*_2_ represent two different wavelengths in micrometres; α represents the Ångström exponent. The α is a good indicator of the aerosol type and provides qualitative information regarding the dominant aerosol size in the atmosphere. A higher α value represents the dominance of fine-mode particles and vice versa for coarse-mode particles [26]. In this study, α was derived from the AERONET measurements for a wavelength pair at 440–675 nm, and the AOD at 550nm was calculated based on the AOD at 440 nm from the AERONET measurements.

### Diffuse radiation fraction

To investigate the effects of aerosols on diffuse radiation, the diffuse radiation fraction was calculated as follows [27]:

$$R_{f}=\frac{R_{d}}{R_{s}}\times 100\%$$ (2)

where R_f_ is the diffuse radiation fraction; R_s_ is the total solar radiation; R_d_ is the diffuse radiation.

## Results

### Spatiotemporal variations in AOD in China

**Fig 2:**
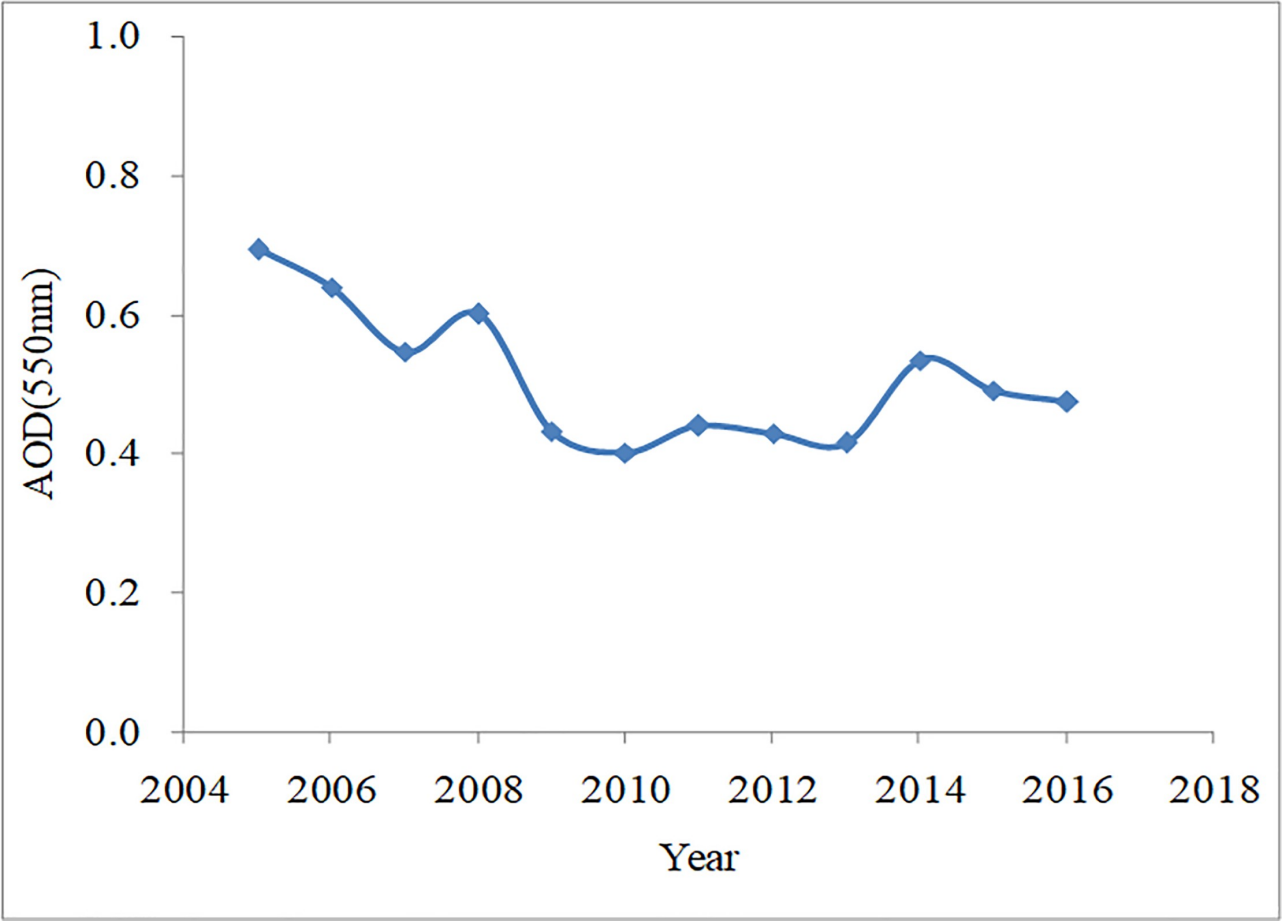
Interannual variation in 550 nm AOD from 2005 to 2016 in China.

The interannual variation in the 550 nm AOD from 2005 to 2016 in China is shown in Fig 2. The 550 nm AOD in China reduced significantly from 2005 to 2016, during which the annual 550 nm AOD fluctuated from 0.36 to 0.69, with an annual average of 0.51. The maximum 550 nm AOD occurred in 2005 (0.69), and the minimum occurred in 2010 (0.40). This shows that, in recent years, air pollution has been alleviated, and air quality has improved.

**Fig 3:**
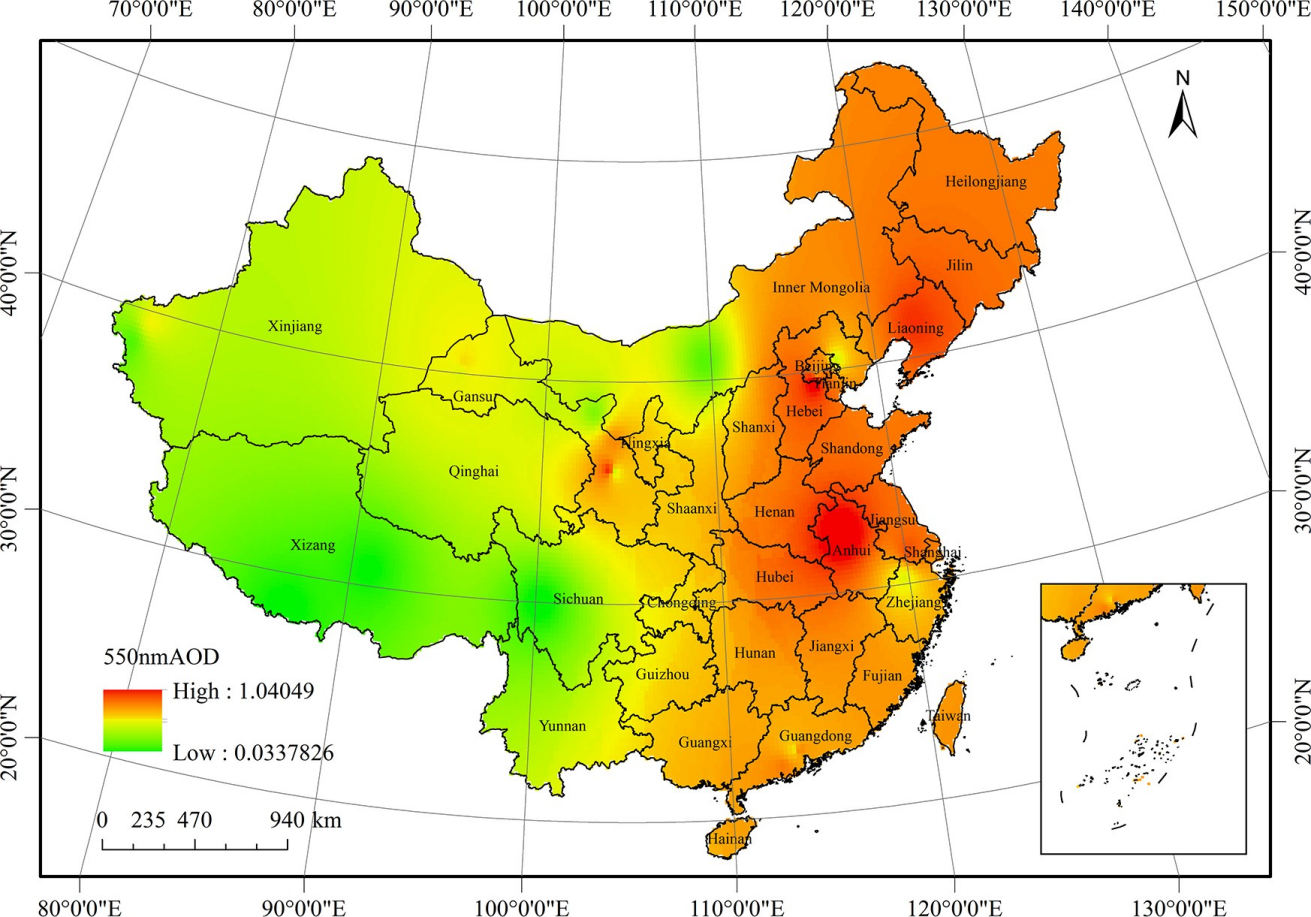
Average distribution of 550 nm AOD in China from 2005 to 2016.

Fig 3 shows the annual average 550 nm AOD distribution in China from 2005 to 2016. This distribution is high in the east and low in the west, with an average of 0.48. Spatially, the average 550 nm AOD fluctuated between 0.03 and 1.09. In particular, the higher AOD areas were the North China, Northeast China, and central China. These areas are densely populated and developed industrially. Thus, the main reason for the high AOD values is aerosol pollution, which is generated by various human activities, such as industrial and agricultural production, construction, and transportation. The low AOD value regions observed in the Yunnan-Guizhou Plateau and Qinghai-Tibet Plateau in western China, may be related to their high altitude and low population density. In general, aerosol particle emissions in the developed eastern and southern regions of China were more serious than those in the western regions.

### Temporal variation characteristics in total radiation, diffuse radiation, and diffuse radiation fraction in China

**Fig 4:**
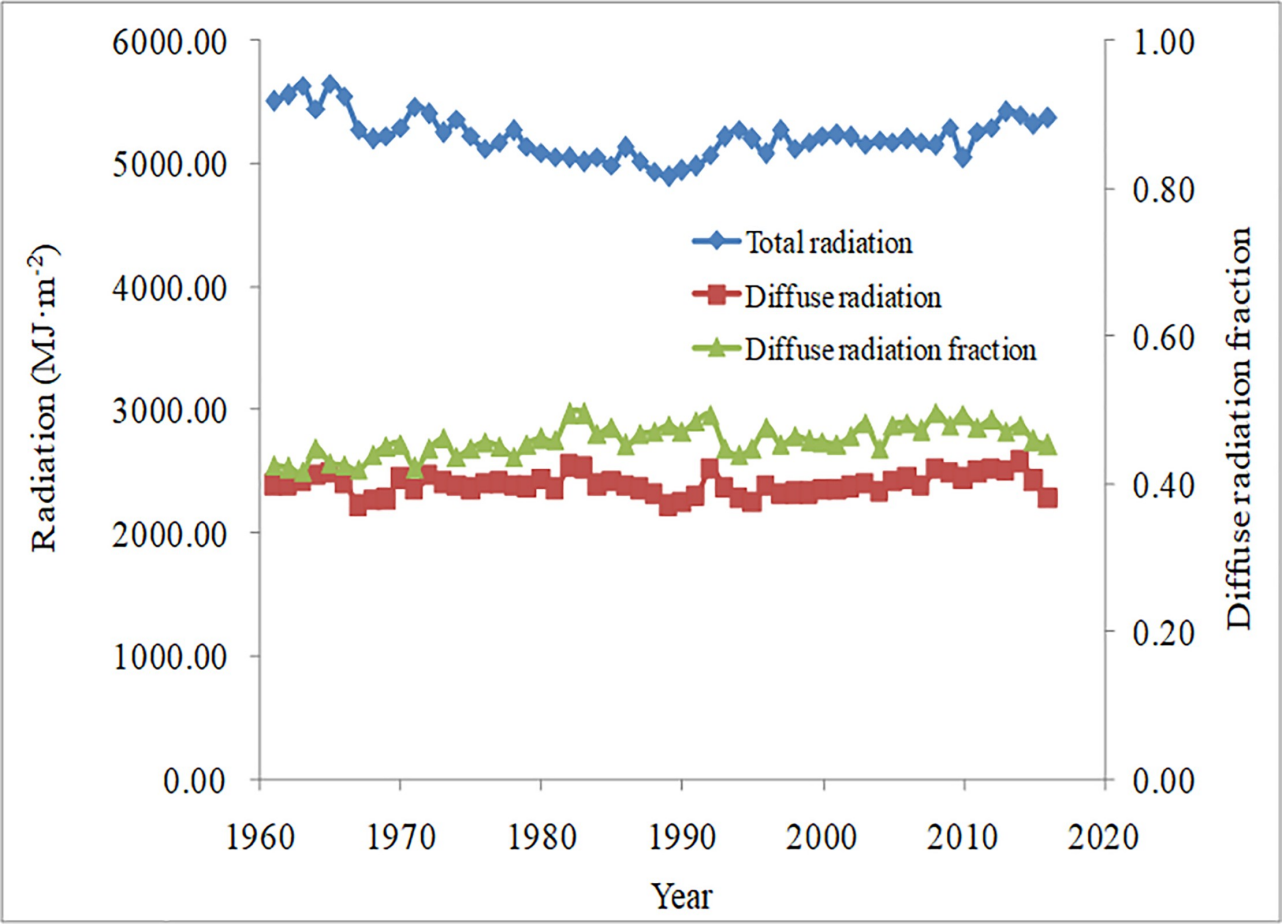
Changes in total radiation, diffuse radiation, and diffuse radiation fraction in China from 1961 to 2016.

The changes in total radiation, diffuse radiation, and diffuse radiation fractions in China from 1961 to 2016 are shown in Fig 4. The average total solar radiation in China from 1961 to 2016 exhibited a downward trend at a decrease rate of 3.10 MJ·m^-2^·yr^-1^, in which the total radiation in the past 56 years decreased by 2.48%. In particular, the total radiation experienced a process of first decreasing and then rising during 1986–1994.

The average diffuse radiation increased slightly from 1961 to 2016 in China, at an increase rate of 3.10 MJ·m^-2^·yr^-1^. Specifically, the diffuse radiation exhibited a downward trend from 1982 to 1989 and then experienced a rapid rise and rapid decline from 1990 to 1995. From the mid-1990s to 2016, the diffuse radiation increased and then decreased. In particular, the average diffuse radiation fraction in China from 1961 to 2016 increased by 6.53%. Meanwhile, from 1992 to 1994, the diffuse radiation fraction experienced a sharp decrease, which might be related to the eruption of the Pinatubo volcano in 1991. After 1994, the diffuse radiation fraction steadily increased with fluctuation.

### Spatial distributions in total radiation, diffuse radiation, and diffuse radiation fraction in China

**Fig 5:**
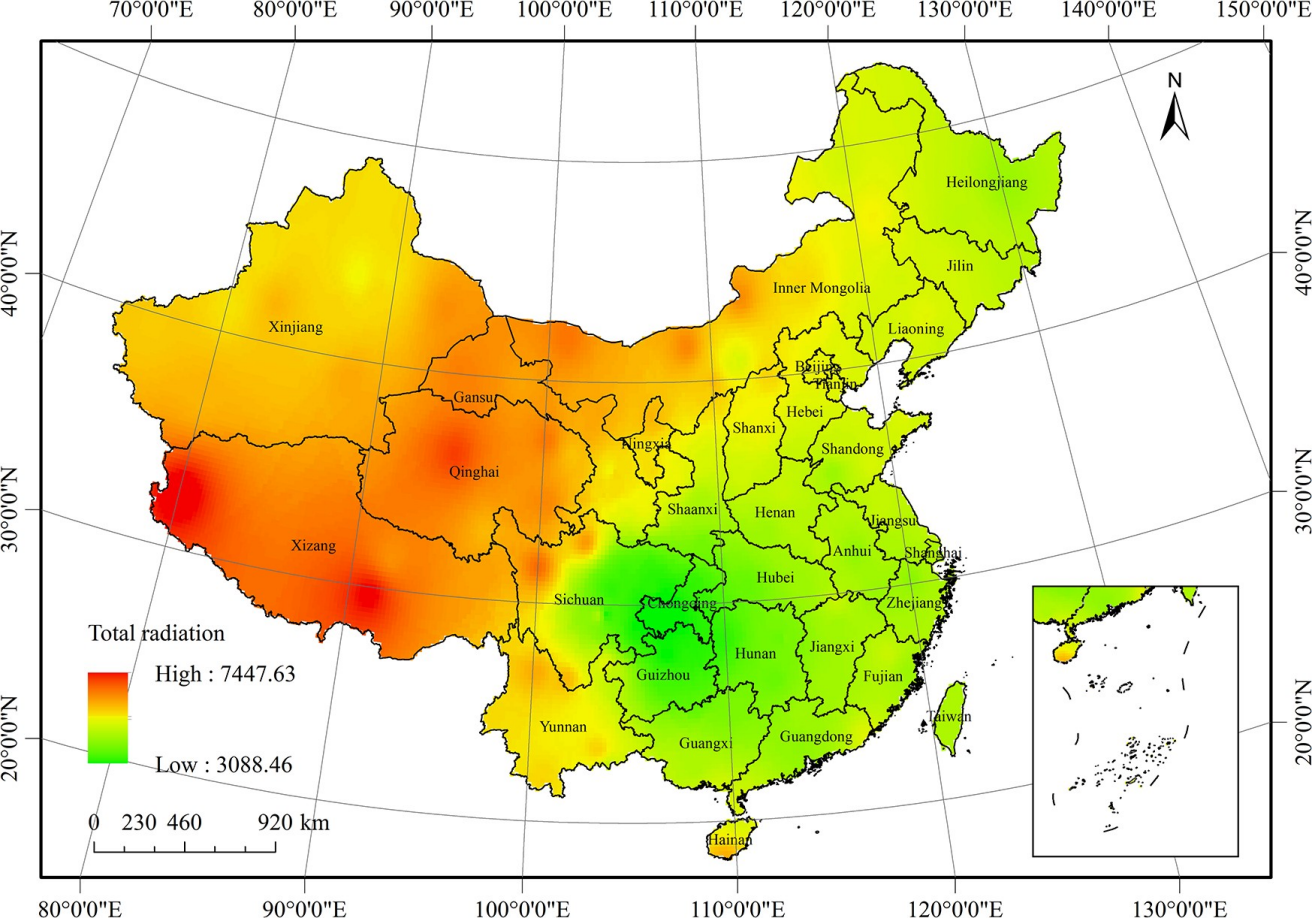
Average distribution of total radiation in China from 1961 to 2016.

The annual average total radiation distribution in China from 1961 to 2016 is shown in Fig 5. Because of the influence of altitude, latitude, and the monsoon climate, the total radiation was unevenly distributed in China, ranging from 3036.5 MJ·m^-2^·yr^-1^ to 7449.4 MJ·m^-2^·yr^-1^, with an average of 5050.2 MJ·m^-2^·yr^-1^. The total radiation in the western plateau was higher than that in the eastern plain. The high-value region of total radiation in China was in the southern Qinghai-Tibet Plateau, in which the total radiation was higher than 6500 MJ·m^-2^·yr^-1^. This was because of the high altitude, thin air, and high transparency of the atmosphere.

**Fig 6:**
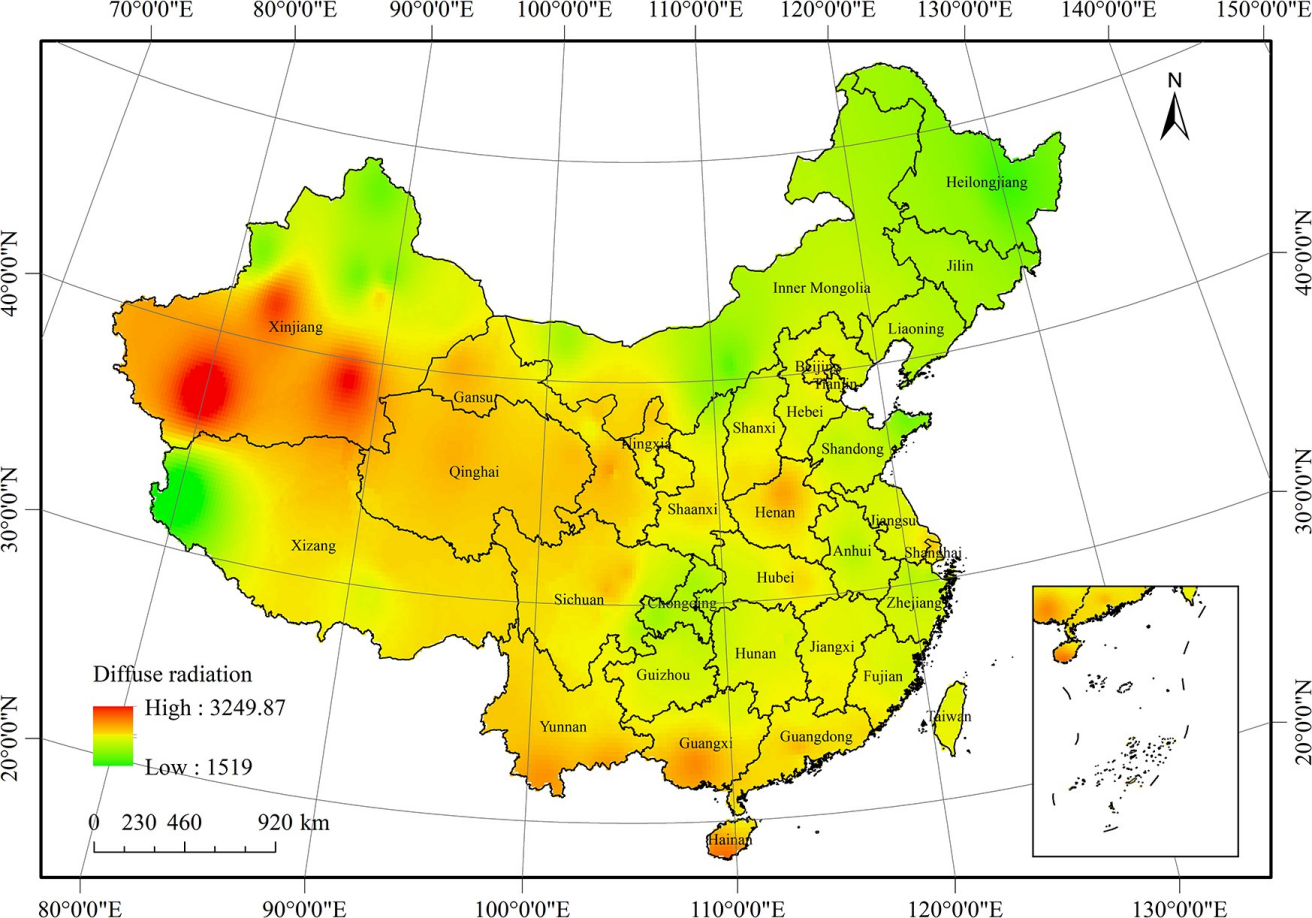
Average distribution of diffuse radiation in China from 1961 to 2016.

The spatial distribution of the average diffuse radiation in China from 1961 to 2016 is shown in Fig 6, varying from 1517.7 MJ·m^-2^·yr^-1^ to 3252.8 MJ·m^-2^·yr^-1^, with an average of 2351.9 MJ·m^-2^·yr^-1^. In general, the diffuse radiation was higher in the south and west and lower in the north. The southern Qinghai-Tibet Plateau and southwestern Yunnan-Guizhou Plateau were high-value centres of diffuse radiation in China, wherein diffuse radiation was higher than 2800 MJ·m^-2^·yr^-1^. Conversely, Northeast China, Inner Mongolia, and Northwest China were low diffuse radiation areas, wherein the diffuse radiation was less than 2300 MJ·m^-2^·yr^-1^.

**Fig 7:**
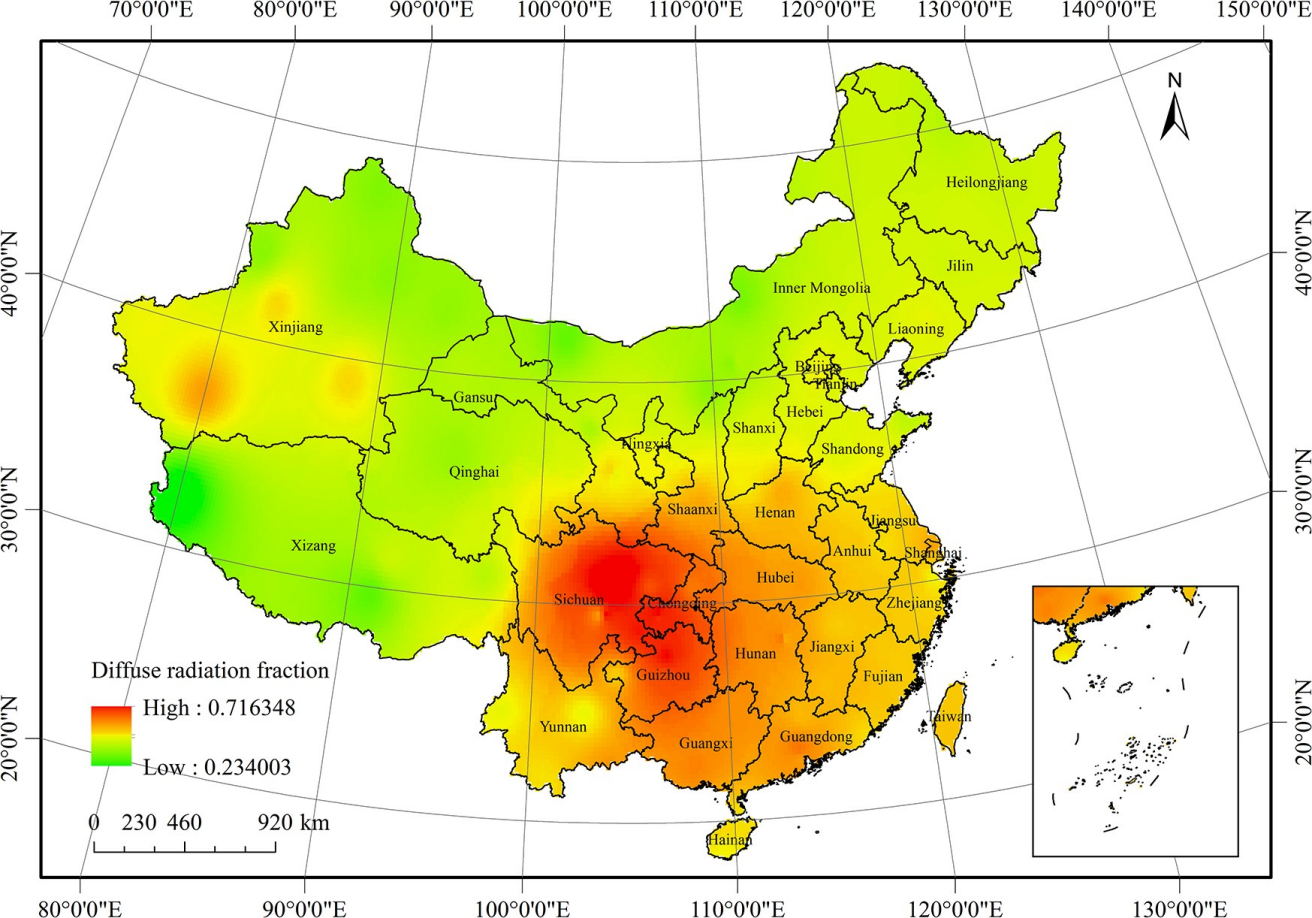
Average distribution of diffuse radiation fraction in China from 1961 to 2016.

The spatial distribution of the annual diffuse radiation fraction in China from 1961 to 2016 is shown in Fig 7. The diffuse radiation fraction in southern China was relatively high. In particular, the Sichuan-Guizhou region and southeast of the Yunnan–Guizhou Plateau were high-value areas, accounting for 71% of the diffuse radiation. Note that from these high value areas, the diffuse radiation fraction decreased gradually in the southeast and northwest directions.

### Influences of aerosol on total radiation, sunshine, and diffuse radiation fraction in China

**Fig 8:**
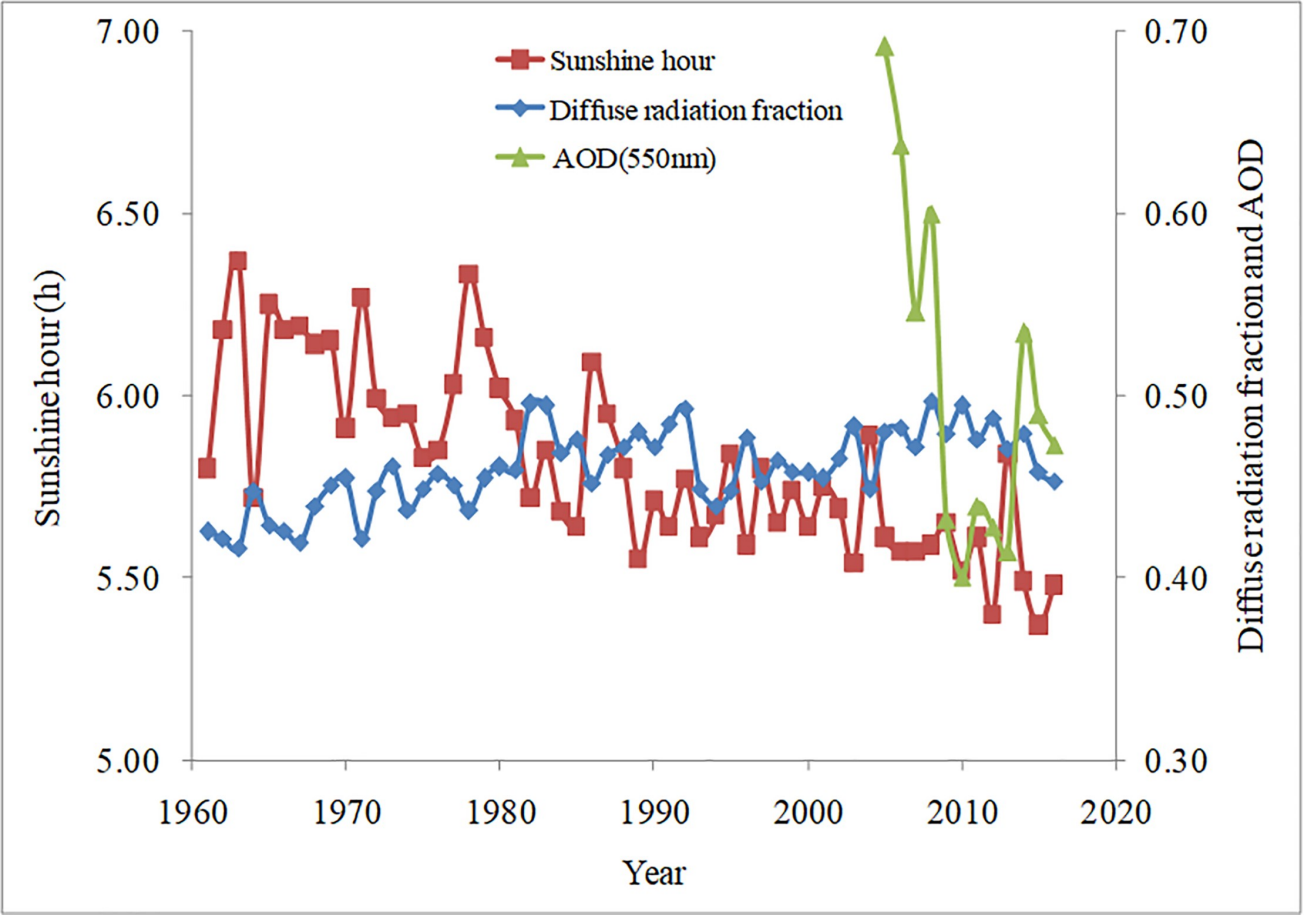
Annual variation of diffuse radiation fraction, sunshine hours, and Aerosol Optical Depth (AOD) from 1961 to 2016.

The relationships among the diffuse radiation fraction, sunshine hours, and 550 nm AOD are shown in Fig 8. The results revealed that the correlations between the diffuse radiation fraction and sunshine hours were negative, while that between the sunshine hours and AOD was positive. Although the AOD data were relatively limited, the interannual variation in the last 10 years revealed a good positive correlation between the diffuse radiation fraction and AOD. Thus, when pollution reduced, the AOD decreased, sunshine hours increased, and the diffuse radiation fraction decreased. Conversely, when pollution was more serious, the AOD and diffuse radiation fraction both increased. In general, over the past 56 years, air pollution in China has become increasingly serious, resulting in a significant reduction in sunshine hours and solar total radiation and an increase in the diffuse radiation fraction.

## Discussion

Many factors can cause the variation of observed solar radiation. In particular, the radiations are altered by changes in the top atmosphere and radiation process reaching the surface [28], such as changes in the solar constant, aerosols impact, and water vapour. However, the effects of different factors on radiation change vary. Currently, of the factors affecting radiation, the clouds and aerosols are focused. Regarding the influence of clouds on radiation, the conclusions obtained in different time ranges and regions are inconsistent. Some scholars believe that cloud cover changes dominate radiation change in China, because the change in total cloud cover is very consistent with radiation change [29], and a strong "darkening" phenomenon has been observed in some areas with low cloud occurrence frequency [2]. This implies that decreases in radiation are caused by increases in anthropogenic aerosol contents, which were consistent with previous observed and simulated results [30–32].

China is the largest developing country in the world, and its rapid economic development and population growth have led to an increasing number of anthropogenic aerosols being discharged into the atmosphere. Under the influence of aerosols, the direct radiation component and diffuse radiation component in total radiation reaching the surface in China have been changed. Studies have found increased haze weather in China with increasing aerosol particles caused by human activities [33], resulting in a lower total radiation and higher diffuse radiation [13, 14, 21, 30, 31]. In our study, we found that aerosols had negative impacts on the total radiation and sunshine hours, but had significantly positive effects on the diffuse radiation and diffuse radiation fraction during the study period, which is, in general, consistency with previous research results [13, 14, 21, 30, 31, 33]. From 1991 to 1993, the total radiation showed a trend of rapid decrease, which may have been related to the eruption of the Pinatubo volcano in 1991. This volcanic eruption released approximately 20 million tons of sulphide into the stratosphere, which immensely reduced the total radiation on the Earth’s surface. The atmosphere gradually recovered after 1994 [22]. Zhang et al. [27] studied the effects of aerosols on radiation by using medium resolution imaging spectrometer products and obtained meteorological data in China from 2000 to 2014. They found that the AOD positively influenced the diffuse radiation and diffuse fraction, and that increasing the diffuse radiation promoted the gross primary productivity of forests.

However, the potential uncertainty in estimating spatiotemporal variations in radiation caused by aerosols in China remains, and might attenuate the results. Regarding spatial data, the interpolation accuracy and quality of radiation and AOD data must be improved in order to obtain accurate evaluation results. In this study, the daily solar radiation data taken from 127 meteorological stations in China between 1961 and 2016 were collected. However, only 80 stations performed simultaneous diffuse radiation observations, and only 34 AERONET sites in China were selected. In addition, the time series length of the AOD data obtained from AERONET was very limited, and comprehensive data from each AERONET station were available beginning in 2005.

Despite these limitations and shortcomings, these results offer a more comprehensive understanding of the spatiotemporal radiation variations caused by aerosols in China from 1961 to 2016. Future researches should further inverse the AOD historical data based on the relationships between solar radiation and AOD in order to obtain the long time series AOD data and provide data support for related research. Nevertheless, aerosol pollutions are not independent climate events, but accompanied by changed cloud amount, reduced sunshine hours, and decreased total solar radiation [34], and thus the mechanism and mutual feedback effects of these factors must be continuously studied.

## Conclusions

The results of this study reveal the spatiotemporal radiation variations caused by aerosols in China from 1961 to 2016. Our results indicate that there were obvious temporal and spatial variations in the AOD, total radiation, diffuse radiation, and diffuse radiation fraction over the past 56 years. Specifically, the 550 nm AOD from 2005 to 2016 decreased significantly, with an annual average of 0.51. Meanwhile, the average total solar radiation in China from 1961 to 2016 decreased by 2.48%, while the average diffuse radiation increased slightly at an increase rate of 3.10 MJ·m^-2^·yr^-1^, and the average diffuse radiation fraction increased by 6.53%. Moreover, spatial heterogeneities of the AOD, total radiation, diffuse radiation, and diffuse radiation fractions occurred. The spatial distribution of the average 550 nm AOD was higher in the east and lower in the west. Thus, aerosol particle emissions in the developed eastern and southern regions of China were more serious than those in the west. The total radiation and diffuse radiation were unevenly distributed. Specifically, they were higher in the western plateau than in the eastern plain. In addition, aerosols had negative effects on the total radiation and sunshine hours, but had positive effects on the diffuse radiation fraction. The correlation between the diffuse radiation fraction and sunshine hours was negative, whereas that between sunshine hours and the AOD was positive. This further illustrates that the aggravation of aerosol pollution in China is one of the main reasons for the increase in the diffuse radiation fraction.

In summary, the results of this study have important implications for improving climate change impact studies in China. For a more complete assessment of spatiotemporal radiation variations caused by aerosols in China, future research should consider additional factors, such as heating differences, energy structure adjustments, and heating technology improvements.

## Supporting information

S1 Data (XLSX)

S2 Data (XLSX)

S3 Data (XLSX)

S4 Data (XLSX)

S5 Data (XLSX)
